# Resting Heart Rate Affects Heart Response to Cold-Water Face Immersion Associated with Apnea

**DOI:** 10.3390/biology12060869

**Published:** 2023-06-15

**Authors:** Krzysztof S. Malinowski, Tomasz H. Wierzba, J. Patrick Neary, Paweł J. Winklewski, Magdalena Wszędybył-Winklewska

**Affiliations:** 1 Department of Neurophysiology, Neuropsychology and Neuroinformatics, Faculty of Health Sciences, Medical University of Gdansk, 80-210 Gdansk, Poland; 2 Department of Physiology, Faculty of Medicine, Medical University of Gdansk, 80-210 Gdansk, Poland; 3 Faculty of Kinesiology & Health Studies, University of Regina, Regina, SK S4S 0A2, Canada; 4 Institute of Health Sciences, Pomeranian University of Slupsk, 76-200 Slupsk, Poland

**Keywords:** cold-water face-immersion test, simulated diving test, heart rate, heart rate variability, healthy individuals, autonomic nervous system

## Abstract

**Simple Summary:**

The body’s response initiated by apnea and cooling the face through contact with cold water consists of the simultaneous activation of the sympathetic and parasympathetic division of the autonomic nervous system (ANS) in the so-called diving response. As a result, the heart rate (HR) slows down, systemic circulation vessels constrict, and blood with its oxygen supply is redistributed to cells that are particularly sensitive to hypoxia. The response is adaptive, preferentially protecting brain tissue from the effects of apnea-induced hypoxia. On the other hand, strong stimulation of the intracardiac vagus nerve can cause bradycardia and even a few seconds of asystole. A possible demonstration that basal HR assessed before face immersion can determine the course of the reflex response of the heart would be of prognostic importance and would open the field for practical applications in the prevention of an unfavorable course of cardiodepressive responses.

**Abstract:**

The regular cardiac response to immersion of the face in cold water is reduction in heart rate (HR). The highly individualized and unpredictable course of the cardiodepressive response prompted us to investigate the relationship between the cardiac response to face immersion and the resting HR. The research was conducted with 65 healthy volunteers (37 women and 28 men) with an average age of 21.13 years (20–27 years) and a BMI of 21.49 kg/m^2^ (16.60–28.98). The face-immersion test consisted of stopping breathing after maximum inhaling and voluntarily immersing the face in cold water (8–10 °C) for as long as possible. Measurements included determination of minimum, average, and maximum HR at rest and minimum and maximum HR during the cold-water face-immersion test. The results indicate a strong relationship between the cardiodepressive reaction of the immersion of the face and the minimum HR before the test, as well as a relationship between the maximum HR during the test and the maximum HR at rest. The results also indicate a strong influence of neurogenic HR regulation on the described relationships. The parameters of the basal HR can, therefore, be used as prognostic indicators of the course of the cardiac response of the immersion test.

## 1. Introduction

Breath-holding, together with the face cooling that typically occurs in freediving, triggers complex reflexes as the so-called ‘dive response’. This response is a cardiovascular reaction that facilitates the survival of the organism in the aquatic environment and in hypoxic conditions. The reflex response is particularly well developed in aquatic mammals such as seals and whales, where it plays an adaptive role. Thanks to this, some terrestrial organisms could repopulate aquatic territories, which was conditional on their adaptation to a long-term stay under the water’s surface. During apnea, there is a decrease in O_2_ partial pressure, an increase in CO_2_ pressure, and an increase in H^+^ concentration in arterial blood [1,2]. These stimuli trigger a reflex from arterial chemoreceptors located in the carotid and aortic bodies and chemodetectors in the central nervous system [3,4]. Additional stimulation of the thermally sensitive sensory endings of the trigeminal nerve, located in the facial skin, especially in the paranasal area, causes the trigemocardial reflex, synergistic with the reflex of arterial chemoreceptors during apnea [5].

The response to diving occurs through the co-activation of the sympathetic and parasympathetic systems [6]. The parasympathetic division mainly innervates the atria and cardiac autorhythmic cells. The result is a slowing down of the depolarization of pacemaker cells and consequently, the time necessary for them to reach the treshold potential is extended. Sympathetic stimulation has an antagonistic effect and causes a positive chronotropic effect. However, parasympathetic innervation has a stronger effect and, consequently, slows down the HR [7]. During an apnea dive, the parasympathetic excitation may reduce the HR below 30 bpm and induce bradycardia [8]. In extreme cases, the activity of the sinoatrial node is reduced or completely inhibited, and the atrioventricular node takes over the function of the main pacemaker. The sympathetic nervous system supplies both atria and ventricles. Sympathetic innervation leads to an increased contractility of heart cells by releasing norepinephrine from the ends of the postganglionic sympathetic fibers. Released norepinephrine may, in some cases, favor the appearance of spontaneous stimulation within the ventricles. Moreover, the sympathetic stimulation leads to a strong constriction of peripheral vessels and a decrease in blood flow to large muscle groups (if the diving reflex is not associated with physical exercise) and abdominal organs, with the simultaneous centralization of circulation and redirection of blood to vital organs such as the brain and the heart itself. Systemic vasoconstriction results in an increase in total vascular resistance and an increase in blood pressure [3].

Cardiodepressive reactions arouse great interest due to the various circumstances initiating them and their often spectacular course being combined with the risk of sudden death. A characteristic feature of the cardiac response to diving is the uncertainty in predicting which individuals are at risk [9,10]. Diving response can be considered as a reflection of the potential of the HR regulatory mechanism in emergency situations, related to the activation of adaptive and defense mechanisms by the body. However, exceeding the regulatory scope is connected with the disturbance of the stability of the internal environment of the system, which may lead to situations that are potentially life-threatening [11]. Such accidents are well documented in divers [9].

Under laboratory conditions, a diving response can be simulated through a cold-water face-immersion test (simulated diving test) [12,13]. It involves breath holding and submerging the face in cold water. The regular response to simulated diving is to slow down the HR [14]. A cardiodepressive response is usually preceded by an increase in HR. The gradual acceleration of the HR is observed several seconds before the start of the face immersion and may continue for several seconds after its initiation. The increase in HR is known as the anticipatory effect, preceding immersion. It is a derivative of emotional arousal, caused by the body’s non-specific reaction to stress [12].

The HR represents the dynamic balance between the intracardiac sympathetic and parasympathetic drive. The sympathetic stimulation increases the HR, while the activity of the parasympathetic division has the opposite effect. The electrocardiographic representation of the cardiac cycle is the distance between the peaks of adjacent R waves, referred as RR intervals (RRi). In the electrocardiogram, successive RRi are not identical, which is related to the neurogenic regulation of the heart rhythm [15]. The physiological basis of the different time of adjacent cardiac cycles is the activity of the ANS and its influence on the depolarization of the sinoatrial node. The HR can be described as the mean RRi over the particular time. Additionally, minimum and maximum HR can be evaluated, which corresponds to the longest and shortest RRi [16].

Central regulatory mechanisms determine the heart rhythm, both at rest and during increased psychomotor activity, during response to environmental factors, or altered metabolic needs. It can be assumed that both resting and non-resting HR are correlated with each other, since both are defined by one and the same individual regulatory mechanism [17]. When examining the dependence of the cardiac response in cold-water face-immersion test on the resting HR, the analysis of heart rate variability (HRV) was used to assess the influence of the ANS on HR and to determine the profile of the neurogenic, extrinsic regulation of the heart [18,19]. This study was designed to determine whether HR under steady-state conditions can be used to predict the cardiac response in situations involving the activation of adaptive mechanisms, such as diving response. In addition, we intend to determine whether minimal HR at rest could provide a faster and easier equivalent of the short-term HRV indices assessing intracardiac parasympathetic activity [20]. A demonstration that selected HR indices, assessed before the immersion test, can approximate the course of the cardiac response, would open the field for practical applications aimed at warning against undesirable cardiodepressive reaction during diving.

We hypothesized an especially strong dependence of the cardiodepressive reaction and anticipatory stimulation of the cold-water face-immersion test on the minimum and maximum HR (respectively) before the attempt. In addition, we believe that the examined relationships are gender-dependent, which may result from different regulatory profiles of the cardiovascular system between women and men. We also wanted to test the hypothesis that minimum HR can predict the cardiodepressive response to cold-water face immersion and that this HR indicator can be treated as an easy-to-calculate alternative to short-term HRV indicators.

## 2. Materials and Methods

This study was conducted with 65 healthy volunteers, including 37 women and 28 men, ranging in age from 20 to 27 years. Volunteers were recruited on the basis of data from their medical history and a health questionnaire. The exclusion criteria included diseases of the respiratory and cardiovascular systems and arrhythmias recorded in the resting electrocardiogram (ECG). Participants were informed about the research procedures and gave their written consent to participate in the study. Body measurements included the following characteristics: height, weight, body mass index (BMI), and percent body water and body fat (Tanita, BC-601, Japan, Tokyo). The study was approved by the Independent Bioethics Committee for Scientific Research at the Medical University of Gdansk (NKBBN/471/2013).

The cold-water face-immersion test consisted of stopping breathing after taking a maximum breath and voluntarily immersing the face in water at a temperature of 8 to 10 °C for as long as possible. Prior to the start of the test, the subject remained in a sitting position with elbows and forearms resting on the table (10 min). At a given signal, the volunteers breathed in maximally, held their breath, and then immersed their face. Before and during the study, ECGs were recorded from bipolar limb leads at a sampling rate of 4 kHz. Acquisition and partial analysis of the data was performed using PowerLab instrumentation (ADInstruments Research System, AdInstruments, Dunedin, New Zealand). The cold-water face-immersion test was coordinated with a physician, an internal medicine specialist, who monitored the ECG online for any abnormal heart rhythm. All study participants completed the tests without any adverse events.

### 2.1. Heart Rate Analysis

An epoch containing 512 RR intervals (RRi) was extracted from the resting ECG recording (10 min sitting rest). The mean resting heart rate (HR_0_), the minimum resting heart rate (MIN_HR_0_), and the maximum resting heart rate (MAX_HR_0_) were calculated from the ECG epoch. MIN_HR_0_ and MAX_HR_0_ corresponded to the longest and shortest RRi of all tested intervals, respectively.

In turn, the description of the cardiac response during face immersion in water also included the determination of the longest and shortest heart cycle, RRi, representing the minimum (MIN_div) and maximum (MAX_div) HR during the test.

### 2.2. HRV Analysis

HRV analysis was used to determine the regulatory determinants of HR in both the time and frequency domains using statistical tests and the Fast Fourier Transform (FFT) algorithm, (Kubios HRV Pro Software, Kuopio, Finland). Determinants such as the standard deviation of NN intervals (SDNN) and total power (TP ms^2^) were used to assess total heart rate variability. For the analysis of cardiac parasympathetic influences, the square root of the mean squares of the differences between consecutive RR intervals (RMSSD) and the percentage of differences between RR intervals that exceed 50 ms (pNN50%) were used. In the frequency domain, short-term variability is described by the power of the spectrum in the high-frequency range (HF ms^2^; 0.15–0.4 Hz). The indicator that describes the long-term variability of the HR is the power of the spectrum in the low-frequency range (LF ms^2^; 0.04–0.15 Hz). In addition, non-linear determinants of HR variability were determined using a Poincaré plot and SD_1_ and SD_2_ indices. The Poincaré plot is a graphical representation of the correlation between successive RR intervals, i.e., RRi_+1_ and RRi.

### 2.3. Statistical Analysis

The subject of the analysis was HR at rest and during the cold-water face immersion associated with apnea. The shortest and longest RR intervals were determined using the functions of the calculation program. Results are presented as mean ± standard deviation (SD). The analysis also concerns the study of relationships between resting HR and cardiac response to the test. Interpretation of results was based on statistical analysis using specific tests depending on the distribution of variables. The distribution of data was tested with the Shapiro–Wilk test, while the comparison of the means of the group of men and women was carried out using the *t*-Student test or the Mann–Whitney test. The Pearson or Spearman tests were used to analyze the correlations. For a more accurate analysis of the relationship between resting HR and cardiac response to face immersion, additional subgroups of volunteers were used depending on their values of resting HR and regulatory determinants of heart rate—HRV. The divisions were based on the median in such a way that groups with HR and HRV values greater or less/equal than the median were created in a comparatively numerical manner. Statistical significance was *p* ≤ 0.05. Calculations were made using the statistical analysis program Statistica 10 (StatSoft Inc., Tulsa, OK, USA)

## 3. Results

### 3.1. Anthropometric Data

Table 1 presents body measurements, including age, body mass, height, body mass index (BMI), and body water and body fat percentages.

### 3.2. Resting Heart Rate

The parameters of the resting HR are presented in Table 2. The mean HR (HR_0_) calculated from 512 consecutive heart cycles was 79.40 ± 11.10 min^−^^1^. The minimum resting HR (MIN_HR_0_) representing the longest heart cycle was 60.90 ± 10.02 min^−^^1^ and was statistically lower in men (57.50 ± 9.90 min^−^^1^) compared to women (63.48 ± 9.44 min^−^^1^). The maximum resting HR (MAX_HR_0_), corresponding to the shortest heart cycle, was 101.18 ± 11.40 min^−^^1^.

### 3.3. Heart Response to Face Immersion in Water

The regular cardiac response to apnea associated with immersion of the face in cold water is a reduction in the HR (Table 3). The minimum HR during the immersion (MIN_div) was 52.66 ± 8.16 min^−^^1^ and was significantly lower in men (49.35 ± 7.73 min^−^^1^ vs. 55.17 ± 7.64 min^−^^1^) than women. The cardiodepressive reaction is preceded by anticipatory stimulation, which is a non-specific reaction to stress. The maximum HR (MAX_div), determined by the shortest RRi, was 112.98 ± 16.23 min^−^^1^ and was higher in women (117.44 ± 16.38 min^−^^1^ vs. 107.09 ± 14.25 min^−^^1^). The cardiodepressive response was also determined by the difference between the mean resting HR before the test (HR_0_) and the minimum HR during face immersion—(%∆MIN_div-HR_0_). In turn, the determinant of the anticipatory increase in HR was the difference between the mean resting HR (HR_0_) and the maximum HR during the test—(%∆MAX_div-HR_0_).

### 3.4. Dependence of Cardiac Response to Face Immersion on Resting HR

Table 4 represents the correlation coefficients for the dependence of the cardiac response during immersion of the face in cold water on the resting HR expressed as the minimum (MIN_HR_0_), maximum (MAX_HR_0_), and average (HR_0_) HR before the test. The highest regression coefficients for all participants were recorded for the relationship MIN_HR_0_-MIN_div: 0.5344 and MAX_HR_0_-MAX_div: 0.6080.

Further analyses showed that the relationship between MIN_div and MIN_HR_0_ was dependent on the resting HR, see Table 5. It was shown that the lower the minimum, maximum, and average HR at rest, the stronger the relationship between the lowest resting HR and the lowest HR during the immersion test (MIN_HR_0_ vs. MIN_div). The highest regression coefficients for this relationship for all participants were recorded at low minimum (MIN_HR_0≤MED._), maximum (MAX_HR_0≤MED._), and mean (HR_0≤MED._) resting HR. They were, respectively, 0.5041, 0.6726, and 0.6710. The greatest difference between women and men was at low values of minimum resting HR (MIN_HR_0≤MED._). In men, the correlation coefficient was 0.7348; in women, 0.2210.

The dependence of the highest HR during face immersion in cold water on the highest HR before the test (MAX_HR_0_ vs. MAX_div) also depended on the resting HR. The higher the minimum, maximum, and average resting HR, the stronger the relationship between MAX_HR_0_ and MAX_div. The highest regression coefficients were recorded when the values of minimum, maximum, and mean HR were higher than their medians—MIN_HR_0>MED._, MAX_HR_0>MED._, HR_0>MED_. It should be noted that the correlations in the group of women were higher compared to the group of men.

Moreover, the research results indicate that the relationship between the minimum HR during the face immersion and the minimum resting HR is fundamentally affected by HRV, reflecting the effects of neurogenic regulation on the HR. The correlation coefficients for the MIN_HR_0_ vs. MIN_div relationship in the context of heart rate variability analysis are presented in Table 6 and Table 7. Research results indicate that people with high intracardiac parasympathetic activity are characterized by a strong dependence of the MIN_div on MIN_HR_0_. This is indicated by high correlation coefficients for the described dependence in people with high (higher than the median) indices of short-term variability, such as rMSSD, pNN50%, HF(m^2^), and SD_1_. A particularly strong relationship between MIN_HR_0_ and MIN_div occurs in men with high values of the rMSSD index (rMSSD_>MED._). The correlation coefficient for this subgroup of men is 0.7883, but it does not differ significantly from the correlation coefficient for the corresponding subgroup of women with rMSSD values higher than the median—0.6514.

On the other hand, our results indicate that people with low intracardiac parasympathetic activity are characterized by a strong dependence of the maximum HR during the cold-water immersion test on the maximum HR at rest. This is indicated by high correlation coefficients for the described relationship in people with low short-term variability indices, such as rMSSD, pNN50%, HF (m_2_), and SD_1_.

A particularly strong relationship between MAX_HR_0_ and MAX_div was observed in women with low values of rMSSD (rMSSD_≤MED_.) and HF (ms^2^) (HF_≤MED._). The correlation coefficients for these subgroups are 0.8108 and 0.8483, respectively, but they do not differ significantly from the correlation coefficients for the corresponding subgroup of men with rMSSD and HF (ms^2^) lower than the median, which are, respectively, 0.6672 and 0.6878.

## 4. Discussion

We investigated the relationship between the cardiac response to cold-water face immersion and the resting HR. There were three new findings from this study: (1) the cardiodepressive response and anticipatory effect of a cold-water face-immersion test is dependent on the resting minimum and maximum HR, respectively; (2) minimum resting HR may be a simple and easy to estimate alternative to indices of short-term HRV in predicting the cardiodepressive response to a cold-water face-immersion test; and (3) the dependence of the cardiac response to a cold-water face-immersion test on the resting HR differs between men and women.

To the best of our knowledge, the relationship between resting HR and the cardiac response to the cold-water face-immersion test has never been studied before. The results of our work indicate that the lower the minimal HR at rest, the more pronounced the cardiodepressive response during the immersion test. The relationship between MIN_HR_0_ and MIN_div is much stronger in volunteers with a slow resting HR and high rates of short-term HRV at rest, which suggests the strong influence of the parasympathetic division on the described dependence. The significant role of MIN_HR_0_ in predicting the cardiodepressive response of the face-immersion test could be explained by its dependence on intracardiac parasympathetic activity. Results of our study showed a close correlation between the MIN_HR_0_ and the short-term variability of HRV.

These results support our earlier work, in which we showed the close relationship between the cardiac response to cold-water face immersion and the intracardiac influences of the ANS using HRV analysis [21]. There was a significant correlation between the cardiac response to the face-immersion test (expressed as minimum and maximum HR during the immersion) and HRV, expressed by time and frequency domain determinants at rest. The most relevant results concerned the relationship of the Min_div with the indices of short-term variability (rMSSD, pNN50%, HF (ms^2^), SD_1_), which was evidence of a close relationship between parasympathetic activity at rest and the maximum slowing down of the HR during the cold-water immersion.

Taking into account the results of the cited study and results of the current research, MIN_HR_0_ may be a simple and easy estimating method alternative to indices of short-term HRV in predicting the cardiodepressive response to a cold-water face immersion.

There is also a report of daily variability of the cardiac response in a simulated diving test (immersion of the face in cold water). It was noticed that the resting HR and the determinants of ANS activity changed with the time of day. Using HRV analysis, the researchers indicated greater intracardiac parasympathetic activity in the morning, which was manifested by increased short-term variability of HRV, slower resting HR, and greater cardiodepressive response to immersion of the face in cold water. On the other hand, a smaller reduction in HR during the immersion was noted at noon and in the evening hours, which was correlated with lower parasympathetic activity and a faster resting HR [22].

HR is the manifestation of the activity of extrinsic regulation of the heart, conditioned by the influence of the parasympathetic system slowing the heart rhythm, and the sympathetic system, with the opposite effect. Mean HR is a statistical measure of the entire data distribution, while the minimum HR is the extreme value of this distribution, which implies that it represents the extreme capabilities of the HR regulation system to maximize slowdown of the HR in a given functional state of the body [20].

Face immersion in cold water and apnea cause a cardiodepressive reaction, reducing the oxygen demand of cardiomyocytes, which is particularly important in the conditions of developing hypoxia. It can, therefore, be assumed that the lower the minimum HR during diving, the more efficient the adaptive mechanisms of the response to diving. Since we have confirmed the close relationship between the minimum HR at rest and the minimum HR during the cold-water face-immersion test, it can be concluded that the minimum HR at rest can be a predictive indicator of a better adaptation of the body to diving. Studies comparing the HRV and resting HR between people with several years of free diving experience and a control group of people without such experience indicate a higher total and short-term HRV and a slower resting HR in divers [23]. It is worth noting, however, that exceeding the regulatory capabilities of the heart rhythm may lead to life-threatening situations associated with strong parasympathetic stimulation which causes a significant HR reduction even with a few seconds’ asystole, as well as partial or complete atrioventricular block [24,25].

The subsequent results of the study show that there is a strong correlation between the maximum HR during diving and the maximum HR at rest (MAX_HR_0_ vs. MAX_div). This means that the higher the HR at rest, the greater the anticipatory effect and the higher the maximum HR in the cold-water immersion test. The correlation coefficient for this relation is 0.6080 for all participants. More detailed results indicate, however, that this relationship is much stronger in those with low rates of short-term variability and high MAX_HR_0_ (lower activity of intracardiac parasympathetic nerves). In all subjects whose MAX_HR_0_ is higher than the median (MAX_HR_0>MED._), the correlation coefficient for MAX_HR_0_-MAX_div relationship is 0.7683.

This initial acceleration of the heart rhythm precedes immersion of the face in the water and lasts for the next several seconds after it starts. The anticipatory effect is a body’s stress response, based on sympathetic stimulation related to the fear of the immersion [3,17]. Particularly high activities of the intracardiac sympathetic innervation preceding the face-immersion test is attributed to adolescents whose attitude to this maneuver leads to high, transient tachycardia [26].

Alternatively, sympathetic stimulation plays an important adaptive role here, consisting of vasoconstriction of selected arterial and venous vessels, which leads to centralization of circulating blood and better perfusion of the heart and brain [2,27,28]. In turn, too strong a stimulation of intracardiac sympathetic activity may be associated with undesirable arrhythmias, such as ventricular extrasystoles or other dangerous cardiac events. The anticipatory effect and the maximal HR during face immersion indicate the degree of sympathetic arousal, and as our research shows, maximum HR at rest may be an indicator of sympathetic activation during the cold-water face-immersion test and the adaptive but also potentially dangerous role associated with it [9,24].

Additional research results from our study indicate a significant difference in the relationship between the cardiac response to face immersion and the resting HR in women and men. The relationship between MIN_HR_0_ and MIN_div is most pronounced in men with low values of MIN_HR_0_ (MIN_HR_0≤MED._). In men, a significant lower minimum HR was noted at rest and during cold-water face immersion, which may indicate a higher contribution of the parasympathetic system to heart rhythm compared to women. On the other hand, a higher correlation coefficient of the MAX_HR_0_–MAX_div relationship was observed in the group of women with high values of MAX_HR_0_ (MAX_HR_0>MED._), 0.7341. What is worth emphasizing is that women were characterized by a significantly higher maximum HR at rest and during immersion compared to men. These dissimilarities between women and men are probably dependent on differences in neurogenic regulation of the heart rhythm and, thus, a different profile of circulatory regulation [29]. Although we did not find statistically significant differences in HRV between women and men, which was used to assess the effect of intracardiac ANS activity on the HR, we did find significant differences in the minimum and maximum HR at rest and during the cold-water face-immersion test, which are indirect markers of sympathetic and parasympathetic activity of the heart rhythm. It should be noted that there is no standardized way to present the cardiac response to immersion of the face in cold water. Lin points out that some authors indicate the HR measured immediately before the start of the test as a reference point. However, it should be noted that the HR immediately before the face immersion is higher due to emotional arousal (anticipative effect) and that the results describing the reduction of HR in response to diving are then abnormally high [30].

The variation in resting HR between women and men may be due to intrinsic properties of the sinoatrial node and the neurogenic regulation of the HR. With simultaneous blockade of intracardiac sympathetic and parasympathetic activity, a higher HR was noted in women. This may indicate intracardiac differences between women and men that are independent of neurogenic control of the HR [31]. In turn, HRV analysis assessing the influence of the ANS on spontaneous sinoatrial node discharges indicates differences in the power spectral density for short- and long-term variability in women and men [29]. Some authors indicate that these differences are the result of the influence of sex hormones on cardiac pacemaker cells and cardiomyocytes and on the system of neurogenic regulation of the heart rhythm [32,33].

This study’s results indicate a higher emotional arousal in women, shortly before and in the initial phase of the face immersion. The anticipatory effect is the result of the body’s non-specific response to stress, which, according to some studies, may differ by gender. For example, studies related to the continuous recording of HR in people performing their daily work duties (work-related stressor) indicated significantly higher mean and maximum HR in women [34]. These observations are consistent with animal studies, where acute stressors revealed different body responses to stress in females and males, which is associated with the activity of sex hormones released during the activation of the hypothalamic–pituitary–gonadal axis (HPG) [35].

However, our research also has some limitations. The volunteers were students of the Medical University of Gdansk that described themselves as being of average physical capacity. Since a direct maximal oxygen consumption (VO_2_ max) test was not performed, we were unable to accurately determine the effect of training and exercise capacity on the studied relationship between the cardiac response to the cold-water face-immersion test and the basal HR at rest. Further research on the impact of physical capacity on the studied relationships is needed. Likewise, the participants were novices and unaccustomed to the dive response. In addition, there are uncertainties regarding the physiological interpretation of the LF index in the frequency domain of the HRV analysis, and therefore, the interpretation of the relationship between the cardiac response to face immersion in cold water and the basal HR in people with high or low LF values raises some concerns. Moreover, the minimum and maximum HR measurements at rest and during the face immersion are extreme values. For this type of data, it is advisable to use the test–retest reliability method, so as to compare whether the tested values will be equal at two distinct occasions. Despite the obvious advantages of such a method, it requires repeating the entire research procedure twice.

## 5. Conclusions

The results presented in the study showed a close relationship between the resting HR and the cardiac response to face immersion in cold water. The parameters of the basal HR can, therefore, be used as prognostic indicators of the course of the cardiac response of a cold-water face-immersion test and, thus, indicate the potential danger associated with the occurrence of serious cardiac events. A large reduction of HR, reflected in the low value of the MIN_div index, may indicate a large influence of the parasympathetic system on HR and, at the same time, provide information about the potential strong inhibitory effect of the parasympathetic system on the physiological pacemakers. The obtained results indicate that the resting HR assessed before face immersion in cold water determines the course of the reflex response of the heart and, thus, may open the field for practical applications in the prevention of an unfavorable course of cardiodepressive responses.

## Figures and Tables

**Table 1:** Anthropometric data for all participants. Values are mean ± SD.

		All (n = 65)			Women (n = 37)			Men (n = 28)			*p* [W vs. M]	
	
Age		21.13	±	1.34	21.03	±	0.91	21.30	±	1.89	0.4700	
Body mass (kg)		65.12	±	11.25	59.25	±	7.18	74.73	±	10.08	0.0000	***
Height (m)		1.74	±	0.09	1.69	±	0.05	1.82	±	0.08	0.0000	***
BMI—body mass index (kg/m^2^)		21.49	±	2.33	20.81	±	2.03	22.62	±	2.39	0.0033	***
%Fat (%)		22.05	±	7.80	23.70	±	5.38	18.76	±	10.64	0.0316	*
%H_2_O (%)		54.28	±	3.93	52.67	±	3.36	57.70	±	2.68	0.0000	***

*—*p* < 0.05; ***—*p* < 0.001.

**Table 2:** Resting HR for all participants. Values are mean ± SD.

		All			Women			Men			*p* [W vs. M]	
	
MIN_HR_0_		60.90	±	10.02	63.48	±	9.44	57.50	±	9.90	0.0159	*
MAX_HR_0_		101.18	±	11.40	103.38	±	12.08	98.28	±	9.92	0.0740	
HR_0_		79.40	±	11.10	81.38	±	11.53	76.78	±	10.11	0.0978	

*—*p* < 0.05.

**Table 3:** Heart rate response to cold-water face-immersion test in healthy men and women. Values are mean ± SD.

		All			Women			Men			*p* [W vs. M]	
	
MIN_div		52.66	±	8.16	55.17	±	7.64	49.35	±	7.73	0.0036	**
MAX_div		112.98	±	16.23	117.44	±	16.38	107.09	±	14.25	0.0098	**
%∆_MIN_div-HR_0_ [%]		−32.95	±	10.86	−31.31	±	11.07	−35.12	±	10.38	0.1634	
%∆_MAX_div-HR_0_ [%]		43.75	±	21.82	45.72	±	21.50	41.14	±	22.36	0.4056	

**—*p* < 0.01.

**Table 4:** Correlation coefficients for the dependance of MIN_div and MAX_div on resting HR.

		MIN_div vs.						MAX_div vs:					
		All		Women		Men		All		Women		Men	
MIN_HR_0_		0.5344	***	0.4283	**	0.5454	**	0.4591	***	0.4729	**	0.3038	
MAX_HR_0_		0.3422	**	0.2497		0.3545		0.6080	***	0.6176	***	0.5136	**
HR_0_		0.3835	**	0.3117		0.3816	*	0.5415	***	0.5987	***	0.3627	

*—*p* < 0.05; **—*p* < 0.01; ***—*p* < 0.001.

**Table 5:** Correlation coefficients for MIN_HR_0_ vs. MIN_div and MAX_HR_0_ vs. MAX_div depending on resting HR.

		MIN_HR_0_ vs. MIN_div						MAX_HR_0_ vs. MAX_div					
		All		Women		Men		All		Women		Men	
MIN_HR_0_	≤MED.	0.5041	*	0.2210		0.7348	**	0.3575	*	0.1931		0.4598	
	>MED.	0.1499		0.0051		0.0021		0.7379	***	0.8266	***	0.4285	
MAX_HR_0_	≤MED.	0.6726	***	0.7319	**	0.7876	***	0.1208		0.0280		0.1496	
	>MED.	0.3517		0.4179		0.1516		0.7683	***	0.7341	***	0.3450	
HR_0_	≤MED.	0.6710	***	0.5923	**	0.6835	**	0.1218		0.2070		0.2464	
	>MED.	0.3361		0.3642		0.0725		0.7401	***	0.7770	***	0.5340	*

*—*p* < 0.05; **—*p* < 0.01; ***—*p* < 0.001.

**Table 6:** Correlation coefficients for MIN_HR_0_ vs. MIN_div and MAX_HR_0_ vs. MAX_div depending on the HRV parameters in the time domain.

		MIN_HR_0_ vs. MIN_div						MAX_HR_0_ vs. MAX_div					
		All		Women		Men		All		Women		Men	
SD_NN	≤MED.	0.1675		0.1733		−0.0077		0.7110	***	0.7255	***	0.6507	*
	>MED.	0.6884	***	0.6416	**	0.5333	*	0.4388	*	0.3869		0.4396	
rMSSD	≤MED.	0.3028		0.3463		0.0812		0.8056	***	0.8108	***	0.6672	**
	>MED.	0.7021	***	0.6514	**	0.7883	***	0.3376		0.2437		0.4083	
pNN50%	≤MED.	0.2986		0.3115		0.1533		0.7862	***	0.7974	***	0.7046	**
	>MED.	0.7710	***	0.5839	*	0.5772	*	0.3119		0.2543		0.3850	

*—*p* < 0.05; **—*p* < 0.01; ***—*p* < 0.001.

**Table 7:** Correlation coefficients for MIN_HR_0_ vs. MIN_div and MAX_HR_0_ vs. MAX_div depending on the HRV parameters in the frequency domain.

		MIN_HR_0_ vs. MIN_div						MAX_HR_0_ vs. MAX_div					
		All		Women		Men		All		Women		Men	
Total Power (ms^2^)	≤MED.	0.1729		0.1554		−0.0077		0.6978	***	0.7084	***	0.6507	*
	>MED.	0.7170	***	0.6715	**	0.5333	*	0.4730	**	0.4653		0.4396	
LF (ms^2^)	≤MED.	0.2092		0.1166		−0.1746		0.6761	***	0.5600	*	0.5240	
	>MED.	0.6748	***	0.7421	***	0.6584	*	0.5029	**	0.6368	**	0.5080	
HF (ms^2^)	≤MED.	0.3226		0.2856		0.0754		0.8318	***	0.8483	***	0.6878	**
	>MED.	0.8000	***	0.7024	**	0.7076	**	0.3531	*	0.2233		0.4216	
SD_1_	≤MED.	0.3028		0.3463		0.0812		0.8056	***	0.8108	***	0.6672	**
	>MED.	0.7021	***	0.6514	**	0.7883	***	0.3376		0.2437		0.4083	
SD_2_	≤MED.	0.2179		0.1839		−0.0077		0.7136	***	0.7325	***	0.6507	*
	>MED.	0.6650	***	0.6128	**	0.5333	*	0.4162	*	0.2812		0.4396	

*—*p* < 0.05; **—*p* < 0.01; ***—*p* < 0.001.

## Data Availability

Not applicable.
